# Autologous fascial slings remain viable at long-term follow-up: a post cystectomy case report

**DOI:** 10.1186/s12894-021-00884-7

**Published:** 2021-09-08

**Authors:** Jacopo Durante, Francesca Manassero, Tiziana Fidecicchi, Alessio Tognarelli, Tommaso Di Vico, Pinuccia Faviana, Cesare Selli

**Affiliations:** 1 Department of Translational Research and New Technologies in Medicine and Surgery, Section of Urology, University of Pisa, via Paradisa 2, 56126 Pisa, Italy; 2 Department of Surgical, Medical, Molecular Pathology and Critical Area, Section of Pathology, University of Pisa, via Roma 67, 56124 Pisa, Italy

**Keywords:** Muscle fascia, Autologous fascial slings, Stress urinary incontinence, Suburethral slings, Case report

## Abstract

**Background:**

Autologous fascial slings (AFS) have been used for a very long time in the treatment of female stress urinary incontinence, but the introduction of synthetic mesh slings placed either retropubicallyor trans-obturator has decreased the need to harvest the autologous rectus muscle fascia, thus reducing invasiveness and operative time. However AFS are still indicated in complicated cases and re-interventions, and the FDA has underlined safety concerns over the use of surgical meshes for the transvaginal repair of prolapsed pelvic organs.

**Case presentation:**

A 76-year-old woman with muscle-invasivebladder cancer underwent radical cystectomy 16 years after retropubic positioning of an autologous rectus muscle fascial sling for SUI, with complete symptom resolution. The sling was easily identified and removed en bloc with the bladder and urethra, providing an opportunity to histologicallyevaluate the autologous fascial graft after its long permanence in the new position. Histopathological examination demonstrated increased fibroblastic proliferation and formation of capillaries. A slight separation and an increased waviness of the connective fibers were both evident. An increased vascularity was also apparent, including transverse vessels, with clusters of vessels. A relative inflammatory reaction was present in over 300 cells/10 HPF. All these characteristics indicated viable connective tissue.

**Conclusions:**

AFS remain a valuable surgical option for both primary and recurrent SUI in women, showing high cure rates and low complications in the long-term. The present case, to the best of our knowledge, presents the longest follow-up period of an autologous rectus muscle fascia placed retropubically and its histological evaluation documents characteristics which support its mechanical strength and viability.

## Background

Synthetic mid-urethral slings (MUS) have become the most frequently used surgical intervention for SUI for women [1, 2]

The original AFS technique was first described over a century ago and consisted in the placement of a strip of rectus muscle fascia at the bladder neck. This approach is durable and effective but may lead to the appearance of voiding problems and de novo storage symptoms [3]. A recent Cochrane review analyzed the outcomes between AFS and MUS finding similar effects in terms of continence but a higher rate of complications for the AFS. This review has two major weaknesses. The follow-up takes place after too short a period making it difficult to draw conclusions about the long-term safety of MUS. The other major weakness is related to the patient groups which were not well randomized regarding the severity of symptoms [4].

However,of the FDA has recently reviewed the literature and has underlined serious safety and effectiveness concernover the use of surgical mesh for the transvaginal repair of pelvic organ prolapse (POP). Patients treated with syntheticmeshes are subject to mesh related complications and sequelae (10% erosion within 12 months of surgery) and re-operation,and, moreover,the use of synthetic tissue does not appear to provide any added benefit when compared to traditional surgery [5]. Within this lively panorama of literature that seeks to re-evaluate autologous slings with respect to synthetic meshes, we have decided to share the result of a case where an autologous sling has had an extremely long survival rate and long-term effectiveness.

## Case presentation

In November 2016 a76-year-old woman underwent radical cystectomy for a muscle invasive bladder cancer (MIBC) in our urology unit. The patient had a history of SUI afterhysterectomy and adnexectomy for an ovarian carcinoma in 1997. SUI was treated with an autologous fascial sling from the rectus muscle in 2000according to McGuire [6] with a complete resolution of the urinary leakage up until September 2016, when the patient returned with urgency and urge incontinence. Cystoscopy was performed, showing multiple papillary lesions at the bladder neck and in the left bladder wall. In October 2016 the patient underwent transurethral bladder resection which revealed a muscle invasive bladder cancer. During the radical cystectomy, performing the section of the urethra, the autologous fascial sling was evidenced and macroscopically it appeared to be extremely well preserved. It was removed en bloc with the bladder and urethra.

**Fig. 1:**
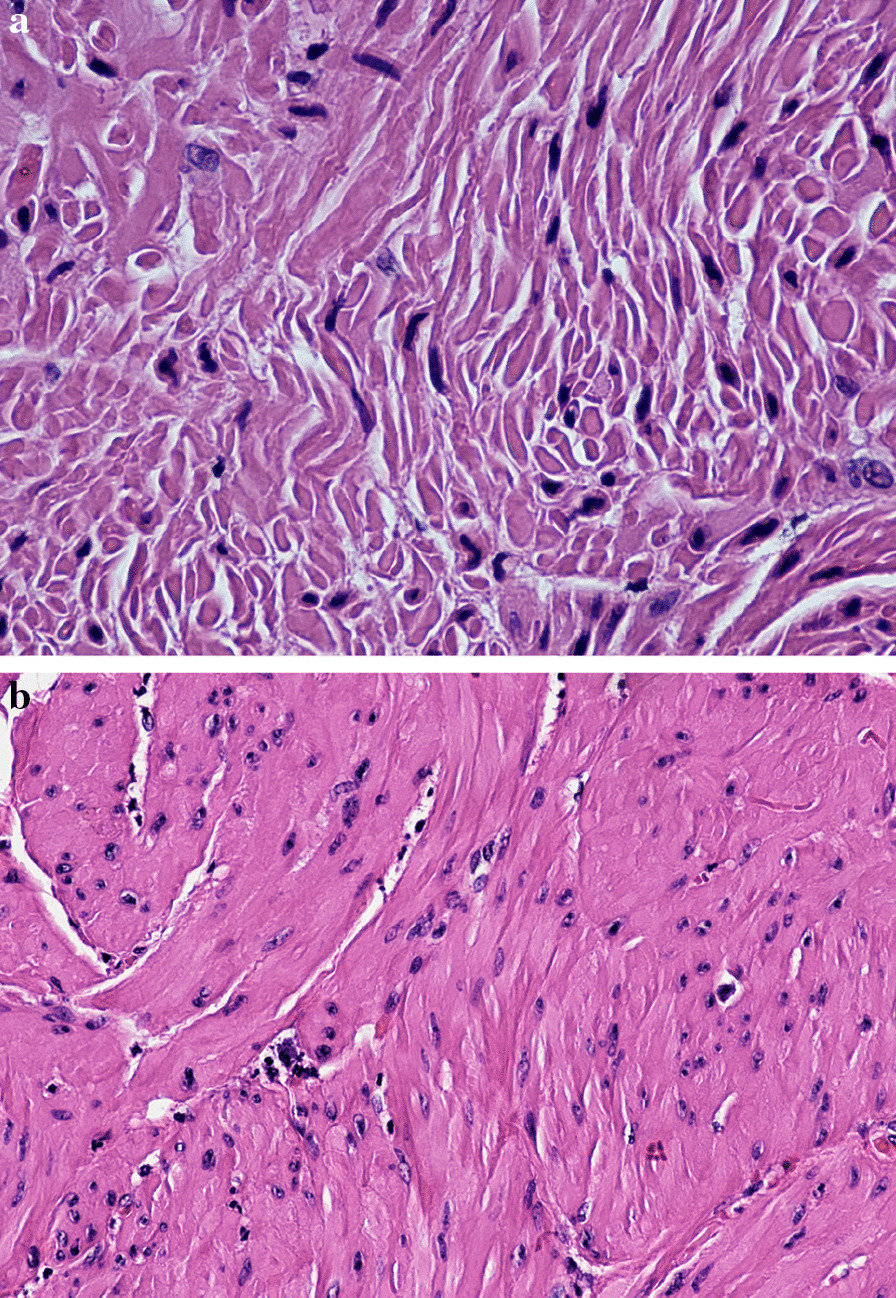
**a** ×20Hematoxylin-eosin: increased fibroblastic proliferation. **b** ×40Hematoxylin-eosin:increased fibroblastic proliferation

**Fig. 2:**
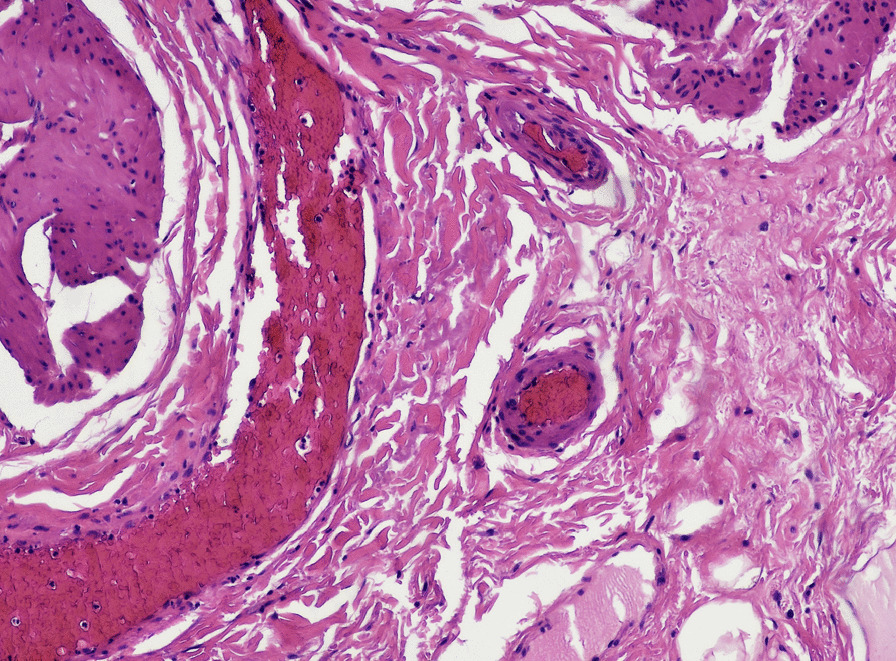
×10Hematoxylin-eosin: increased capillary formation and modest inflammatory reaction consisting mainly of lymphocytes and plasma cells

The entire surgical specimen was fixed in 10% neutral-buffered formalin. The bladder neck, urethra and sling were isolated, embedded in paraffin and sectioned at 4–5 mm intervals, according to routine procedures. Histologic sections were stained with hematoxylin and eosin to evaluate fiber structure, the number of cells and vascularity. Increased fibroblastic proliferation was evident (Fig. 1), as well as a slight separation of fibers and increased waviness. There was increased vascularity, including transverse vessels relative to thefiber direction, with over 300 cells/10 HPF, with clusters of vessels (Fig. 2), and a modest increase in inflammatory infiltrate consisting mainly of lymphocytes and plasma cells.

## Discussion

Since their introduction, syntheticmid-urethral slings have become the most commonly used surgical intervention for the female SUI, since they require shorter intraoperative and hospitalization times as well a shorter learning curve [3–5]. A recent systematic review by Rehman et al. evaluated the difference between the traditional suburethral sling and other treatments for the mixed urinary incontinence. They found that rectus fascia slings have a better outcome in the first year of follow-up than other biological materials (porcine dermis, lyophilized dura mater, fascia lata and vaginal wall). However, in the review they reported more side effects with the traditional slings than the minivasiveones, when compared to a similar success rate (although the quality of the evidence was very variable in the various studies analyzed). The traditional sling seems to have a better cost-effective rate in comparison to other SUI treatments but not in comparison tosynthetics [7, 8].

Many studies analyzing the outcomes of synthetic mesh lack a long-term follow-up. A recent multicenter trial by Khan et al., however, found that even if there is not enough evidence to judge the long-term success rates between autologous and synthetic sling there is however some evidence that the “dry rate” for an autologous sling could be more durable. In the long term the AFS could be considered at least equivalent to TVT but with a higher success rate after mesh complication [9].

The histological evaluation of the sample taken from our patient demonstrates the scarcity of studies availableon the characteristics of the material used in autologous slings in the long-term. The behavior over time of biological tissues used for reconstructive treatment could be the basis for the success or failure of the application of an autologous sling. Fitzgerald et al. [10] removed non- functioning or obstructive suburethral AFS, between 5 and 18 weeks after placement, and removed only one after a period of 4 years. The latter showedincreased vascularityat histopathology, with connective tissue fibers organized longitudinally.

Animal models can provide useful information on how to integrate autologous tissues in the treatment of SUI or on which they can be most effective. McAdams et al. hypothesized that there may be a benefit in combining an autologous fascial graft and a deltoid flap into murine models [11].

Autologous fascial graft, according to Carvalho et al. seems not to cause inflammatory reaction response in the histologic evaluation on grafted fascia in vocal fold of dog harvested two months from its implantation [12].

The vitality of autologous grafted tissue many years after its implantation is not yet well known. Ahelen et al. hypothesized that there may be a regeneration and normalization, after a long period, of the semitendinosus tendon used for the reconstruction of the cruciate ligament. After an average of 8.4 years, they did not detect significant differences in the histological appearance between the regenerated tissues and those of the contralateral knee that had not been harvested [13].

The greater awareness of the side effects related to the use of synthetic mesh has also made way for stem cells in the treatment of SUI or other diseases in which autologous tissues or synthetic implants are used. In a phase-one clinical study, Choi et al. evaluated the efficacy of a periurethral injection of adipose-derived autologous stem cells. The assessment was based on questionnaires related to continence but also on more objective methods (the functional length of the urethra evaluated with MRI and the measure of maximum urethral closing pressure) and evidenced better continence without indicating serious side effects [14]. Orabi et al. managed to organize cells from the vaginal epithelium and vaginal stroma to form a neovagina that was guided to assemble on a three-dimensional matrix and then implanted in vivo. This study on mouse models shows how an autologous tissue can be used to create a vagina which is functionally and biologically similar to a natural one without using external materials [15].

What is interesting is the search for new surgical techniques which will be able to reduce the invasiveness of autologous slings. In a recent paper Osman et al. have illustrated a new andless invasive technique in which the autologous sling is positioned at the mid-level of the urethra and not at the level of the bladder neck. The midurethral placement of the graft is advantageous since it requires a lesser bladder neck dissection (thus avoiding the bladder base denervation and so lessening risk of detrusor overactivity and storage urinary symptoms), hematoma, bladder injury and fibrosis. This measure would allow patients with primary SUI to be treated with autologous material, thus extending the current indications which use the autologous sling mainly for patients with intrinsic sphincter deficiency or in the recurrences of SUI after a synthetic sling application. An additional benefit is that the learning curve of this technique is relatively short (approximately 20 procedures) [16].

Woodruff et al. evaluated 24 grafts of different origin (polypropylene mesh(PPM), autologous fascia, porcine dermis, cadaveric dermis, cadaveric fascia) removed between 2 and 34 months after implantation. They demonstrated a more marked graft degradation in autologous and cadaveric fascia grafts. The PPM had the greater number of fibroblastic infiltrations but neovascularity was mostly represented in AFS [17]. The histopathological analyses of our ASF highlighted the fibrotic and inflammatory reaction: peripheral infiltration of fibroblasts and the relative inflammatory response, consisting of lymphocytes and plasmacells.There were no signs of degradation. In our opinion, this is strongly linked to the clinical efficacy reported by the patient even after such a long interval after the implant. The AFS degradation finding in Woodruff's study could therefore be linked to the fact that these slings were no longer functional. Therefore a biological degradation of the autologous graft can correspond to a reduction of its efficacy from a clinical point of view. However, it is not known whether other well-functioning AFSs retain histological characteristics of integrity in the long-term. This data would be a useful tool for assessing the long-term reliability of this type of implant.

## Discussion and conclusions

The renewed interest in autologous fascial slings in literature is supported both by its long-term efficacy on the symptoms and the reduced need for additional surgical adjustment and mesh-related compliances.

The present case report aims to sustain this new trend of thought and to focus the attention on the autologous sling.

In addition, an autologous fascial sling causes less fibrotic reaction than the synthetic, thus making future surgical treatments easier for SUI and other diseases.

For commercial reasons the evolution of synthetic meshes has been extremely rapid and, since they are associated with a simpler implant technique, they have become the most frequently adopted surgical solution in the treatment of SUI. As a result, this has hindered the evolution and study of autologous tissues and their properties as possible grafts.

The present histologic documentation of the preserved viability and strength of an autologous rectus fascia after being in use for 16 years provides further proof of the viability of this solution in the treatment of SIU.

## Data Availability

The patient charts are available at the AOUP centralized archives and the histopathological materials at Pisa University Division of Pathology.

## Abbreviations

AFS: Autologous fascial slings
HPF: High power field
SIU: Stress urinary incontinence
TVT: Transvaginal
